# Revealing subthreshold motor contributions to perceptual confidence

**DOI:** 10.1093/nc/niz001

**Published:** 2019-02-18

**Authors:** Thibault Gajdos, Stephen M Fleming, Marta Saez Garcia, Gabriel Weindel, Karen Davranche

**Affiliations:** 1 Aix Marseille University, CNRS, LPC, Marseille, France; 2 Wellcome Centre for Human Neuroimaging, University College London, London, UK; 3 Max Planck UCL Centre for Computational Psychiatry and Ageing Research, University College London, London, UK; 4 Aix Marseille University, CNRS, LNC, Marseille, France

**Keywords:** perception, confidence, motor processes

## Abstract

Established models of perceptual metacognition, the ability to evaluate our perceptual judgements, posit that perceptual confidence depends on the strength or quality of feedforward sensory evidence. However, alternative theoretical accounts suggest the entire perception-action cycle, and not only variation in sensory evidence, is monitored when evaluating confidence in one’s percepts. Such models lead to the counterintuitive prediction that perceptual confidence should be directly modulated by features of motor output. To evaluate this proposal here we recorded electromyographic (EMG) activity of motor effectors while subjects performed a near-threshold perceptual discrimination task and reported their confidence in each response in a pre-registered experiment. A subset of trials exhibited subthreshold EMG activity in response effectors before a decision was made. Strikingly, trial-by-trial analysis showed that confidence, but not accuracy, was significantly higher on trials with subthreshold motor activation. These findings support a hypothesis that preparatory motor activity, or a related latent variable, impacts upon confidence over and above performance, consistent with models in which perceptual metacognition integrates information across the perception-action cycle.

## Introduction

Our perception of the outside world is associated with variable degrees of confidence. For instance, when driving in fog, we are less confident about the presence of oncoming cars and may slow down accordingly. Prominent computational models, grounded in signal detection theory and evidence accumulation frameworks, suggest that perceptual confidence is determined by the strength of an internal decision variable that encodes evidence in support of different interpretations of a stimulus (Vickers 1979; Galvin *et al.* 2003; Kiani and Shadlen 2009). Intuitively, the stronger the perceptual evidence, the more confident we should be in our decision. Such models in turn predict that perceptual discrimination accuracy and confidence should be tightly coupled, and be affected by similar experimental manipulations.

However, recent theoretical models of metacognition suggest that the entire perception-action cycle, and not only variation in sensory evidence, may be monitored when evaluating confidence in one’s decisions (Pouget *et al.* 2016; Fleming and Daw 2017). Such models lead to the counterintuitive prediction that confidence in perceptual judgements should be specifically modulated by features of motor output as well as perceptual input. Indeed, recent observations suggest that confidence in perceptual tasks does not only result from feedforward sensory input, but also depends on other sources of information (Siedlecka *et al.* 2016, 2018a,b). For instance longer response times (Kiani *et al.* 2014) or unexpected arousal (Allen *et al.* 2016) have been found to modulate confidence without affecting accuracy. Confidence in perceptual tasks is also disrupted by manipulation of movement speed (Palser *et al.* 2018) and transcranial magnetic stimulation (TMS) of the motor system (Fleming *et al.* 2015). However, a link between trial-by-trial motor output and perceptual confidence remains to be established.

Here, we set out to study this relationship by recording subthreshold fluctuations in electromyographic (EMG) activity as sensitive markers of motor preparatory activity during perceptual discrimination. Specifically, partial muscular activations (henceforth, partial activations) corresponding to subthreshold motor responses have repeatedly been observed in between-hand choice reaction time tasks (Eriksen *et al.* 1985; Hasbroucq *et al.* 1999; Meckler *et al.* 2017). These partial activations are typically followed by an error-related negativity (ERN) in the electroencephalographic (EEG) signal (Scheffers *et al.* 1996; Vidal *et al.* 2000; Meckler *et al.* 2017), and intracerebral recordings demonstrate that the ERN is elicited in supplementary motor area (SMA) and pre-SMA/rostral cingulate (Bonini *et al.* 2014), thereby establishing a direct link between a component of motor output (partial activation) and frontal lobe structures involved in performance monitoring and metacognition (Carter *et al.* 1998; Coull *et al.* 2016; Fleming *et al.* 2018). Notably, variations in error-related EEG potentials have also been recently linked to subjective confidence in perceptual decisions (Boldt and Yeung 2015). Taken together, these findings suggest a hypothesis in which partial activations are specifically associated with modulation of retrospective confidence but not task performance (there is no prior evidence that partial activations impact discrimination accuracy).

Here, we recorded EMG activity of response agonists while participants performed a difficult perceptual discrimination task by pressing the appropriate key with the left or right thumb. After each trial, they were required to verbally provide their confidence in their response. We hypothesized that, consistent with models in which confidence integrates information across the perception-action cycle, partial activations might impact upon confidence over and above performance.

## Results

### Overview of the methods

**Figure 1.**
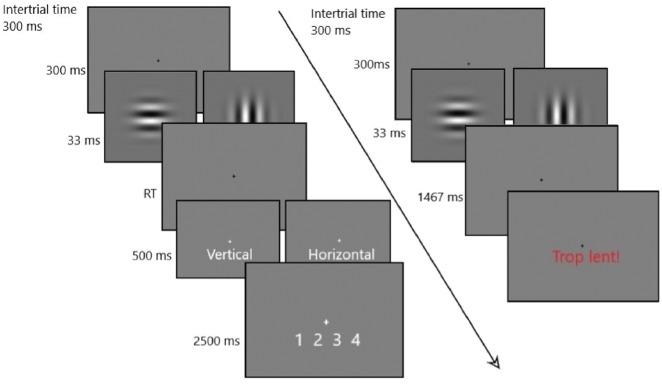
Trial sequence. Successfully completed trial (left). A fixation point was displayed for 300 ms. A Gabor patch (shown in the image) briefly appeared (33 ms) followed by another fixation point. Subjects were required to respond as quickly and accurately as possible according to whether the stimulus was oriented vertically or horizontally by pressing the appropriate key in <1500 ms. Following the response an answer confirmation was displayed for 500 ms. Subjects then had 2500 ms to verbally rate their confidence on a scale from 1 (low confidence) to 4 (high confidence). Failed trial (right). If a response was not provided in <1500 ms, the French words “Trop lent!” (“too slow”) were displayed, and the next trial began after 300 ms.

We analysed data from 19 participants (see Materials and Methods for details) who performed a perceptual discrimination task described in Fig. 1. On each trial, a low-contrast grating was presented centrally for 33 ms. Participants were instructed to indicate, as quickly as possible, whether the grating was oriented vertically or horizontally by pressing the appropriate response key with the right or the left thumb. During each trial, EMG activity of the ‘flexor pollicis brevis’ of each thumb and force production were recorded. After making their decision, subjects were asked to rate their confidence verbally on a scale from 1 (low confidence) to 4 (high confidence). Each subject performed 699 trials evenly split into 10 blocks separated by self-paced rest periods. During a preliminary calibration block, stimulus contrast was manipulated with a 1-up-3-down staircase procedure expected to yield 80% correct responses. On each trial, participants were also allowed to report if they consciously detected making an error. These trials (3.3% of the total number of trials) were omitted from further analysis.

**Figure 2.**
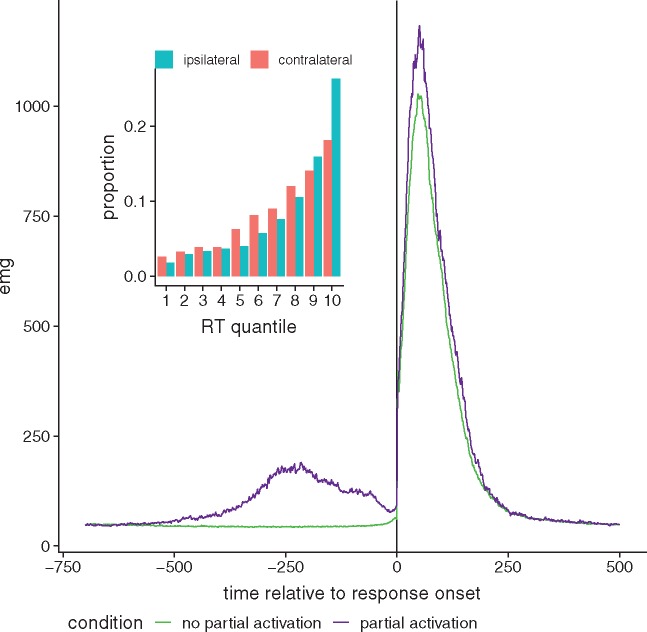
Main figure: Average EMG time course for trials with (purple) and without (green) partial activation. Trials were aligned to response onset (time = 0). Inset figure shows proportion of partial activation [ipsilateral (blue) and contralateral (red) to the response] by RT quantile, averaged across subjects.

EMG traces were inspected off-line, trial by trial, as displayed on a computer screen and the onsets of the changes in activity were hand-scored. After visual inspection, 4.3% of the trials were rejected because of tonic activity or artefacts. Trials with more than one partial activation (5.7% of the trials) were discarded from further analysis. Remaining trials (11 471, mean = 604 per subject, SD = 64) were classified into three categories: trials without partial activation (mean = 83%, SD = 0.09), trials with a partial activation ipsilateral to the provided response (mean = 8.5%, SD = 0.05) and trials with a partial activation contralateral to the provided response (mean = 8.3%, SD = 0.06). The average time course of EMG for trials with and without partial activation is shown in Fig. 2. Reaction time (RT) was measured between the onset of the stimulus and the onset of the required motor response. As expected, partial activations were more frequent among the slowest trials, on which there is more time for them to occur (mean RT for trials without partial activation = 626 ms, SD = 144 ms; mean RT for trials with ipsilateral partial activation = 810 ms, SD = 187 ms; mean RT for trials with contralateral partial activation = 755 ms, SD = 174 ms). Mean accuracy for the orientation discrimination was 80% (SD = 11%), and mean confidence was 2.6 (SD = 0.32).

### Results: impact of partial activations on confidence and accuracy

#### Confidence

We analysed the effects of partial activation on confidence within a hierarchical linear mixed-effects model, including the intercept, partial activation (separately according to whether it is ipsilateral or contralateral to the response), response accuracy, force production and reaction time as fixed and random effects (see Materials and Methods for details). Both reaction time and force production were entered as covariates. Results of the regression analysis are reported in Table 1. As expected, confidence was higher for correct versus error trials (*β* = 0.60, SE = 0.055, *P* < 0.001) and faster versus slower responses (*β* = −2.3, SE = 0.18, *P* < 0.001) (Henmon 1911; Baranski and Petrusic 1998; Kiani *et al.* 2014). Crucially, there was also a significant effect of partial activation on confidence: participants reported higher confidence for trials with compared to without partial activation. Interestingly, this effect was similar for ipsilateral (*β* = 0.15, SE = 0.040, *P* = 0.002) and contralateral (*β* = 0.16, SE = 0.049, *P* = 0.004) partial activations (effect size of ∼0.15 confidence SD). Thus, while partial activations were associated with a significant effect on confidence, the congruency between partial activation and response had no effect on confidence. Finally, confidence was also higher when responses were provided with stronger force (*β* = 0.33, SE = 0.11, *P* = 0.007).

**Table 1.** Hierarchical regression coefficients predicting confidence from accuracy, ipsilateral and contralateral partial activations, reaction time and force production

Predictor	*β*	*P*
Intercept	2.1*** (0.12)	<0.001
Accuracy	0.60*** (0.055)	<0.001
Reaction time	−2.3*** (0.18)	<0.001
Force production	0.33** (0.11)	0.007
Ipsilateral	0.15** (0.040)	0.002
Contralateral	0.16** (0.049)	0.004

Predictors were coded as follows—Accuracy: error = 0, correct = 1; Ipsilateral: absent = 0, present = 1; Contralateral: absent = 0, present = 1.

* *P* < 0.05,

** *P* < 0.01,

*** *P* < 0.001. Reaction time and force production are median-centred. Number of subjects: 19. Number of observations: 11 471.

**Figure 3.**
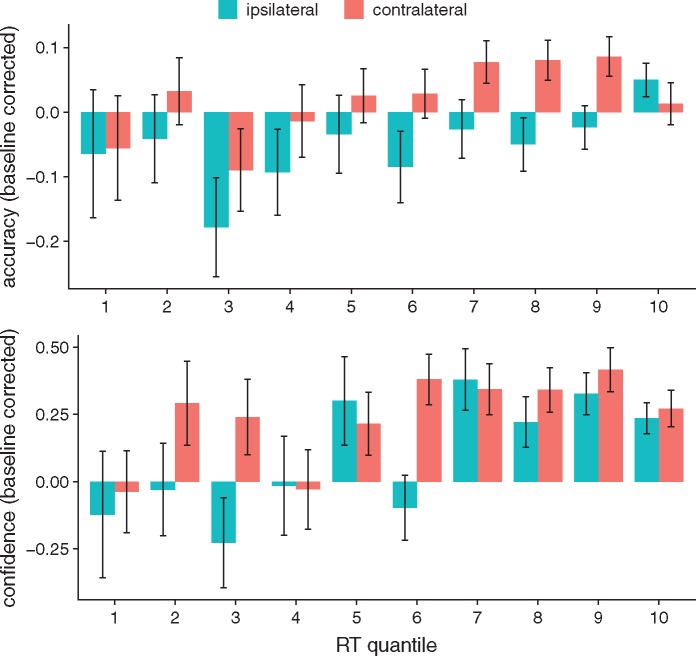
Mean accuracy (top panel) and confidence (bottom panel) by RT quantiles across subjects with partial activation ipsilateral (blue) or contralateral (red) to the response, baseline-corrected with respect to trials without partial activation. Error bars reflect standard errors of the mean.

To further understand the drivers of the partial activation effect on confidence, we visualized confidence and accuracy data as a function of partial activation and response time (Fig. 3). The lower panel of Fig. 3 illustrates a consistent boost in confidence within a majority of RT quantiles on trials with partial activations. Interestingly, inspection of Fig. 3 indicated that the effect of partial activations on confidence effect was most prominent for the slowest RT quantiles, suggesting an interaction between RT and partial activation.

To formally test for such an effect, we replicated the previous analysis, now including an interaction term between RT and partial activation (complete results are presented in Supplementary Table S1.1). We indeed found a significant interaction between RT and contralateral partial activations (*β* = 0.40, SE = 0.17, *P* = 0.004), with a similar trend for ipsilateral activations (*β* = 0.50, SE = 0.26, *P* = 0.07). An interaction between RT and partial activations might be explained by fewer partial activations among fast trials, resulting in a lack of sufficient statistical power to detect their potential effect on confidence. Alternatively, it might be partial activations become a cue to confidence specifically on longer trials (see Supplementary Fig. S1.1). The current data do not allow us to decide between these two explanations.

#### Accuracy

We analysed the effects of partial activation on accuracy within a hierarchical linear mixed-effects model, including the intercept, partial activation (ipsilateral or contralateral), as well as force production and reaction time as fixed and random predictors. Again, reaction time and force production were entered as covariates. Results of the regression analysis are reported in Table 2. Accuracy was higher for faster responses (*β* = −2.3, SE = 0.40, *P* < 0.001). Importantly, however, partial activations, either ipsilateral or contralateral, were unrelated to accuracy on a trial-by-trial basis (all *P*s > 0.35). Force production also had no significant effect on accuracy (*P* = 0.49). We reasoned that a null result of partial activation on accuracy could be attributed to a lack of statistical power for this analysis. However, it is difficult to provide a precise answer to this issue, as we do not have a clear idea of what effect size we should expect for the impact of partial activations. Post hoc power analysis based on 5000 simulations using the simR package in R (Green and MacLeod 2016) reveals that an effect size of −0.4 (observed effect size: *β* = −0.11) for ipsilateral partial activations would have had an 80% chance of being detected in our study at the 5% level. Likewise, an effect size of 0.4 (observed effect size: *β* = 0.14) for contralateral partial activations would have had a 76% chance of being detected.

**Table 2.** Hierarchical regression coefficients predicting accuracy from ipsilateral and contralateral partial activations, reaction time and force production

Predictor	*β*	*P*
Intercept	1.8*** (0.22)	<0.001
Reaction time	−2.3*** (0.40)	<0.001
Force production	0.13 (0.19)	0.49
Ipsilateral	−0.11 (0.15)	0.44
Contralateral	0.14 (0.15)	0.35

Number of subjects: 19. Number of observations: 11 471.

Predictors were coded as follows—Ipsilateral: absent = 0, present = 1; Contralateral: absent = 0, present = 1.

* *P* < 0.05,

** *P* < 0.01,

*** *P*< 0.001.

As for confidence, visual inspection of Fig. 3 suggests a possible interaction effect between RT and partial activations on accuracy. Including an interaction term between RT and partial activations, we found a negative main effect of ipsilateral activations (*β* = −0.40, SE = 0.16, *P* < 0.01), qualified by a positive interaction with RT (*β* =  1.2, SE = 0.47, *P* = 0.009) such that the fastest responses were associated with worse accuracy. No main effects (*β* = −0.22, SE = 0.14, *P* = 0.13) or interactions with RT were found for contralateral activations (*β* = −0.32, SE = 0.62, *P* = 0.61) (see Supplementary Table S1.3 and Supplementary Fig. S1.2).

## Discussion

Recent studies suggest that the motor system contributes to perceptual confidence above and beyond perceptual evidence (Fleming *et al.* 2015; Palser *et al.* 2018). It has also been shown that partial muscular activations of response effectors in between-hand choice reaction time tasks are typically followed by an error-related negativity (Ne) in the EEG signal (Scheffers *et al.* 1996; Vidal *et al.* 2000; Meckler *et al.* 2017) elicited in SMA and pre-SMA/rostral cingulate (Bonini *et al.* 2014). Taken together, these findings led us to design a pre-registered experiment postulating that partial muscular activation of response effectors would have a specific impact on perceptual confidence, over and above any effect on first-order performance (Saez Garcia *et al.* 2017). We remained agnostic about the direction of this effect (i.e. whether partial activations would boost or reduce confidence).

Our results give support to our main hypothesis. More precisely, we found that subjects systematically report higher confidence levels for trials with ipsilateral or contralateral partial activations. Notably, the results for first-order performance were quite distinct to that on confidence: we observed a negative relation between ipsilateral (but not contralateral) activations and accuracy, together with a positive interaction with response time. This pattern suggests that partial activations, or some internal variable covarying with them, have independent influences on confidence, over and above their impact on accuracy. Because accuracy and confidence do not covary in the presence of partial activations, one might wonder whether partial activations also impair metacognitive sensitivity. We tested for this assumption by measuring the impact of the average number of partial activations across trials on individual metacognitive efficiency, measured by the meta-d’/d’ ratio (Maniscalco and Lau 2012) implemented in R by the metaSDT package (Craddock 2018). The results of an OLS regression did not reveal any significant evidence of a relation between partial activations and metacognitive efficiency (*β*= −0.34, SE = 0.63, *P *=* *0.60). This negative result might of course be due to the small number of trials available with partial activations and the likely small size effect (if any), and should thus be taken with caution.

Interestingly, it has been shown that bounded accumulation models predict that higher within-trial evidence variability may lead to increases in confidence and reductions in accuracy (Zylberberg *et al.* 2016), thereby generating a pattern close to that obtained for ipsilateral partial activations in the current study. It might thus be tempting to conjecture that partial activations simply reflect a more volatile sensory signal. Given that we used near-threshold, briefly presented grating stimuli, we are unable to directly evaluate evidence variability in the current design. However, the bounded accumulation model also predicts that more noisy evidence induces faster decisions. Thus, if partial activations were to reflect more volatile information, they should correspond to faster trials. In fact here we observe the opposite (see Fig. 2): responses are slower for trials with partial activation (mean = 780 ms, SD = 184 ms) than for trials without partial activation (mean = 626 ms, SD = 144 ms). In other words, the relation between partial activations, accuracy and confidence appears not to be explained by the strength of the sensory evidence (which would have a parallel impact on both accuracy and confidence), nor by its variability.

While we are unable to exclude that partial activation might be driven by some other component of the sensory signal that simultaneously influences confidence but not accuracy, because partial activations occur well before the decision, a causal impact of subthreshold motor activations on confidence seems plausible. An alternative hypothesis is that such “early” effects of motor activation may indicate that they influence metacognitive evaluation in the same manner as faster responses, acting as a cue to higher confidence. Indeed, it has been shown that response times directly affect confidence levels in perceptual discrimination (Kiani *et al.* 2014). Under this explanation, we should observe an impact of the timing of partial activations on confidence, with earlier activations corresponding to higher confidence levels. To test this hypothesis, we repeated our previous analysis, adding the onset time of the first muscular activation (absolute pre-motor time, Apmt) as a predictor in the regression. Note that in the absence of partial activation, Apmt corresponds to the earliest detectable time of the actual response. In opposition to an explanation based on response timing, shorter Apmt values had no significant impact on confidence level (*β =* −0.19, SE = 0.22, *P* = 0.39). Therefore, the influence of partial activation on confidence cannot be explained by the fact that they occur earlier than the final response (see Supplementary Table S1.2). This suggests that partial activation represents a distinct influence on confidence, rather than another marker of faster responding.

Our results build on and extend previous studies suggesting that the motor system itself contributes to metacognitive judgements in decision-making (Fleming *et al.* 2015; Siedlecka *et al.* 2016, 2018a,b; Palser *et al.* 2018). However, our study represents the first to demonstrate a specific association between subthreshold motor activation—as measured using EMG—and perceptual confidence.

It should be stressed that the link between partial activation and confidence was independent of the congruence of the provided response: subjects consistently reported higher confidence in trials where either ipsilateral or contralateral partial activations were present. This is in line with a recent study showing that subjects’ stimulus awareness in a discrimination task (which is presumably related to perceptual confidence) is increased when they are required to provide a lateralized motor response unrelated to the task before answering (Siedlecka *et al.* 2018a). In contrast, it has been previously shown that single-pulse TMS increases confidence when applied to the hemisphere associated to the chosen response, but decreases it when applied to the hemisphere associated to the alternative response (Fleming *et al.* 2015). One potential account of these seemingly conflicting results is that partial motor activations and pre-motor TMS affect confidence judgements through distinct mechanisms. It has been shown that partial activations are followed by a ERN (Meckler *et al.* 2017) originating in SMA proper (Bonini *et al.* 2014), a structure involved in performance monitoring (Coull *et al.* 2016). It might thus be the case that partial activations are interpreted as efficiently corrected errors, leading to higher confidence in the final decision. In contrast, stimulation of lateralized pre-motor cortex is more likely to directly influence the representations associated with a particular response, and therefore influence confidence in an asymmetric manner. Our data do not provide the means to directly test this explanation, and further research, potentially combining motor TMS with measurement of partial activation, is needed to understand the relation between these effects.

In summary, our findings contribute to growing evidence that confidence in perceptual decisions not only depends on feedforward sensory evidence, but also depends on motor information. They also suggest that different components of motor output might contribute differently to confidence. Together our findings are broadly consistent with “second-order” models in which metacognitive confidence integrates information across the perception-action cycle (Fleming and Daw 2017). We cannot rule out that partial activations are caused by a latent sensory variable that simultaneously and independently boosts confidence—although we are not aware of a model along these lines that would be consistent with our results. Much remains to be done to understand how exactly these components are combined and processed into metacognitive judgements.

## Methods and Materials

### Participants

Sample size was based on previous published study investigating the role of pre-motor activation on confidence (Fleming *et al.* 2015). We decided prior to data collection to ensure data analysis would be possible for between 16 and 25 participants. A total of 27 participants with normal or corrected to normal vision and no history of neurological or psychiatric disorders thus participated in the study. Five participants were excluded before analysis either because the EMG signal was too noisy (two participants), they did not comply with the instructions (two participants) or they failed to accomplish the task (one participant). Furthermore, three outliers were excluded because they displayed multiple activations in >70% of the trials (two participants), or used the highest level of confidence in >80% of the trials (one participant). We thus analysed data of 19 participants in the final sample (11 females, 7 males; mean age = 23 years, SD=3.7). We note that results are qualitatively similar if outliers are not excluded (see Supplementary Appendix 2). Data will be made available on Open Science Framework.

### Stimuli and procedure

The experiment was carried out at the Laboratoire de Neurosciences Cognitives, in Marseille (France). Participants performed the experiment in a dark and sound-shielded Faraday cage. They were seated 100 cm in front of a 15-inch CRT monitor with a refresh rate of 60 Hz. Response buttons were be fixed on the top of two plastic cylinders (diameter 3 cm, height 7.5 cm), 20 cm apart. Participants responded by exerting at least 600 mg of pressure on a button. Responses were confirmed by audio feedback.

Stimuli were generated using the Psychopy library for Python (Peirce 2009). Each trial started with a fixation cross, displayed for 300 ms. It was followed by a low-contrast grating randomly oriented either horizontally or vertically, and presented centrally for 33 ms (4° diameter). Participants were instructed to indicate, as quickly as possible, whether the grating was vertically or horizontally oriented by pressing the appropriate response key with their right or left thumb. The association between grating orientation (horizontal or vertical) and response key (left or right) was counterbalanced across subjects. Responses faster than 1500 ms were confirmed by a message reporting the provided answer (horizontal or vertical) displayed for 500 ms. Responses longer than 1500 ms were omitted from analysis and feedback was provided to the subject signalling that her answer was too slow. After confirmation of the response, subjects were asked to loudly rate their confidence from 1 (low confidence) to 4 (high confidence) while a scale ranging from 1 to 4 was displayed on the screen for 2500 ms. If they consciously detected making a mistake they were asked to say “error” instead of their confidence level. Responses were recorded and written down by the experimenter, who was outside the room where the participants sat. Each trial lasted 4333 ms.

For each participant, stimulus contrast was individually adjusted in a preliminary session using a 1-up-3-down staircase procedure. The experiment consisted of 699 trials, split into nine blocks of 70 trials each and one block of 69 trials. Rest periods between each block were self-paced.

### EMG and force recording

EMG activity was recorded continuously from pre-amplified Ag/AgCl electrodes (Biosemi^®^ Active-Two electrodes^®^, Amsterdam), pasted onto the skin of the thenar eminence over the ‘flexor pollicis brevis’ of each thumb, about 2 cm apart. The common reference electrode was situated above the first vertebra. The signal was filtered and digitized online (bandwidth: 0–268 Hz, 3 dB/octave, sampling rate: 1024 Hz). The experimenter continuously monitored the signal and asked subjects to relax their muscles if the signal became noisy.

The thumb force production was measured as a force signal and digitized on line (A/D rate 2 kHz) allowing us to record the force applied by the participant and trigger a response signal when a force threshold of 600 g is exceeded.

### Data analysis

No statistical analyses were conducted prior to having completed the collection of data for all participants. The protocol was registered on Open Science Framework before starting the experiment (Saez Garcia *et al.* 2017).

#### EMG analysis

The recorded EMG signals were analysed off-line. The trace corresponding to the EMG was displayed on the computer screen and the EMG onsets were hand-scored. Importantly, at this stage the experimenter was unaware of the confidence scores registered separately, avoiding any bias in analysis. Reaction time was measured between the onset of the stimulus and the required motor response. We also defined Absolute Pre-Motor Time (Apmt) as the delay between the onset of the stimuli and the beginning of the first muscular activation.

#### Trials exclusions

Force production was not registered for 50 trials (0.4% of the total number of trials), due to the force peak latency occurring >1500 ms following stimulus onset. After visual inspection, 715 trials (5.4% of the total number of trials) were discarded from further analysis due to the presence of tonic activity or artefacts. Trials reported as errors by subjects were also disregarded (442 trials, 3.3% of the total number of trials). Finally, trials with more than one partial activation were excluded in initial analyses (779 trials, 5.9% of the total number of trials), but were included in the regression analysis reported in Tables S1.2, S2.3 and S3.3. In our primary analyses, we excluded a total of 1810 trials (13.6%), and analysed 11 471 trials (mean = 604 per subject, SD = 64).

#### Statistical procedure

The influence of partial activation on confidence and accuracy was analysed with hierarchical linear and generalized mixed-effects models, respectively, using the lmer4 package (Bates *et al.* 2015) in R (version 4.2, R core Team 2017). All regressions were performed with the restricted maximum likelihood fitting method, and *P* values for coefficients were obtained with the lmerTest package (Kuznetsova *et al.* 2017). We used an alpha level of 0.05 for all statistical tests.

Predictors were coded using treatment contrasts (thus “accuracy” takes a value of 1 if the trial is correct, and 0 otherwise, “ipsilateral” takes a value of 1 if there is a partial ipsilateral activation, and 0 otherwise; “contralateral” takes value of 1 if there is a partial contralateral activation, and 0 otherwise). Reaction time and Absolute Pre-Motor Time (Apmt) were measured in seconds. Force was measured in kilograms. Reaction time, Absolute Pre-Motor Time and force are median-centred. To keep models tractable, we assumed zero correlation between random effects.

We checked that results were robust to the inclusion of outliers (Supplementary Appendix 2) and we replicated all analyses using Bayesian methods as implemented in the Brms package in R (Supplementary Appendix 3) (Bürkner 2016).

## Funding

S.M.F. is supported by a Sir Henry Dale Fellowship jointly funded by the Wellcome Trust and the Royal Society (206648/Z/17/Z)

*Conflict of interest statement.* None declared.

## Supplementary Material

Supplementary Data
