# Supplementary material for: Revealing subthreshold motor contributions to perceptual confidence
Source: Neurosci Conscious. 2019 Feb 18;2019(1):niz001. doi: 10.1093/nc/niz001 (PMC6379662; doi:10.1093/nc/niz001)
Supplement: Supplementary Data [file niz001_supp.docx]

# Appendix 1: Supplementary material

Table S1.1: Hierarchical regression coefficients predicting confidence from accuracy, ispilateral and contralateral partial activations, reaction time, force production and the interactions between partial activations and reaction time. Predictors were coded as follows – Accuracy: error = 0, correct = 1; Ipsilateral: absent = 0, present = 1; Contralateral: absent = 0, present = 1. **^*^**p < .05, ^**^p < .01, ^***^p< .001.

| Predictor | β | p |
| --- | --- | --- |
| Intercept | 2.1^***^ (.12) | < .001 |
| Accuracy | .59^***^ (.055) | < .001 |
| Reaction time | -2.4^***^ (.18) | < .001 |
| Force production | .32^**^ (.10) | .007 |
| Ipsilateral | .071 (.044) | .13 |
| Contralateral | .12^*^ (.050) | .026 |
| Reaction time x Ipsilateral | .50 (.26) | .071 |
| Reaction time x Contralateral | .49^**^ (.17) | .004 |

Number of subjects: 19. Number of observations: 1147

Figure S1.1: *Visualisation of interaction between RT and partial activation. Estimated coefficient for the impact of ipsilateral (left panel) and contralateral (right panel) partial activations on confidence. Shaded region represents 95% confidence. Inset figures show the distributions of ipsilateral and contralateral partial activations onset times.*

Table S1.2: Hierarchical regression coefficients predicting confidence from accuracy, ispilateral and contralateral partial activations, absolute pre-motor time (Apmt), force production and the interactions between partial activations and reaction time. Predictors were coded as follows – Accuracy: error = 0, correct = 1; Ipsilateral: absent = 0, present = 1; Contralateral: absent = 0, present = 1. **^*^**p < .05, ^**^p < .01, ^***^p< .001.

| Predictor | β | p |
| --- | --- | --- |
| Intercept | 2.1^***^ (.12) | < .001 |
| Accuracy | .59^***^ (.055) | < .001 |
| Reaction time | -2.2^***^ (.23) | < .001 |
| Apmt | - .19 (.22) | .39 |
| Force production | .33** (.11) | .007 |
| Ipsilateral | .12^**^ (.045) | .009 |
| Contralateral | .14^*^ (.055) | .02 |

Number of subjects: 19. Number of observations: 11471

Table S1.3: Hierarchical regression coefficients predicting accuracy from ispilateral and contralateral partial activations, reaction time, force production and the interactions between partial activations and reaction time. Predictors were coded as follows – Accuracy: error = 0, correct = 1; Ipsilateral: absent = 0, present = 1; Contralateral: absent = 0, present = 1. **^*^**p < .05, ^**^p < .01, ^***^p< .001.

| Predictor | β | p |
| --- | --- | --- |
| Intercept | 1.8^***^ (.22) | < .001 |
| Reaction time | -2.4^***^ (.41) | < .001 |
| Force production | .12 (.19) | .52 |
| Ipsilateral | -.40^*^ (.16) | .011 |
| Contralateral | .22 (.14) | .13 |
| Reaction time x Ipsilateral | 1.2^**^ (.47) | .009 |
| Reaction time x Contralateral | -.32 (.62) | .61 |

Number of subjects: 19. Number of observations: 1147

Figure S1.2: *Visualisation of interaction between RT and ipsilateral activation. Estimated coefficient for the impact of ipsilateral partial activations on accuracy. Shaded region represents 95% confidence. Inset figure show the distribution of ipsilateral partial activations onset times.*

# Appendix 2: Replication without excluding outliers

Table S2.1: Predicting Confidence from Accuracy, Ispilateral and Contralateral partial activation, Reaction time and Force production. Predictors were coded as follows – Accuracy: error = 0, correct = 1; Ipsilateral: absent = 0, present = 1; Contralateral: absent = 0, present = 1. **^*^**p < .05, ^**^p < .01, ^***^p< .001.

| Predictor | β | p |
| --- | --- | --- |
| Intercept | 2.1^***^ (.13) | < 0.001 |
| Accuracy | .71^***^ (.095) | < 0.001 |
| Reaction time | -2.2^***^ (.16) | < 0.001 |
| Force production | .35^**^ (.096) | .002 |
| Ipsilateral | .11^**^ (.039) | .009 |
| Contralateral | .13^**^ (.044) | .009 |

Number of subjects: 22. Number of observations: 12645

Table S2.2: Predicting Confidence from accuracy, ispilateral and contralateral partial activation, reaction time, force production and the interactions between partial activations and reaction time. Predictors were coded as follows – Accuracy: error = 0, correct = 1; Ipsilateral: absent = 0, present = 1; Contralateral: absent = 0, present = 1. **^*^**p < .05, ^**^p < .01, ^***^p< .001.

| Predictor | β | p |
| --- | --- | --- |
| Intercept | 2.1^***^ (.13) | < 0.001 |
| Accuracy | .70^***^ (.095) | < 0.001 |
| Reaction time | -2.3^***^ (.17) | < 0.001 |
| Force production | .34^**^ (.093) | .002 |
| Ipsilateral | .040 (.040) | .33 |
| Contralateral | .097^*^ (.044) | .04 |
| Reaction time x Ipsilateral | .54^*^ (.22) | .02 |
| Reaction time x Contralateral | .49^**^ (.15) | .001 |

Number of subjects: 22. Number of observations: 12645

Table S2.3: Hierarchical regression coefficients predicting confidence from accuracy, ispilateral and contralateral partial activations, absolute pre-motor time (Apmt) and force production. Predictors were coded as follows – Accuracy: error = 0, correct = 1; Ipsilateral: absent = 0, present = 1; Contralateral: absent = 0, present = 1. **^*^**p < .05, ^**^p < .01, ^***^p< .001.

| Predictor | β | p |
| --- | --- | --- |
| Intercept | 2.1^***^ (.13) | < .001 |
| Accuracy | .70^***^ (.095) | < .001 |
| Reaction time | -2.1^***^ (.21) | < .001 |
| Apmt | -.12 (.20) | .55 |
| Force production | .35** (.095) | .002 |
| Ipsilateral | .093* (.041) | .03 |
| Contralateral | .11* (.048) | .03 |

Number of subjects: 22. Number of observations: 12645

Table S2.4: Hierarchical regression coefficients predicting accuracy from ispilateral and contralateral partial activations, Reaction time and force production. Predictors were coded as follows – Ipsilateral: absent = 0, present = 1; Contralateral: absent = 0, present = 1. **^*^**p < .05, ^**^p < .01, ^***^p< .001.

| Predictor | β | p |
| --- | --- | --- |
| Intercept | 1.7*** (.21) | <.001 |
| Reaction time | -2.2^***^ (.39) | < .001 |
| Force production | .29 (.19) | .13 |
| Ipsilateral | -.17 (.13) | .20 |
| Contralateral | .21 (.14) | .12 |

Number of subjects: 22. Number of observations: 12645

Table S2.5: Hierarchical regression coefficients predicting accuracy from ispilateral and contralateral partial activations, Reaction time, force production and the interactions between partial activations and reaction time. Predictors were coded as follows – Ipsilateral: absent = 0, present = 1; Contralateral: absent = 0, present = 1. **^*^**p < .05, ^**^p < .01, ^***^p< .001.

| Predictor | β | p |
| --- | --- | --- |
| Intercept | 1.7*** (.21) | <.001 |
| Reaction time | -2.3^***^ (.40) | < .001 |
| Force production | .28 (.19) | .14 |
| Ipsilateral | -.40^**^ (.13) | .003 |
| Contralateral | .27^*^ (.12) | .026 |
| Reaction time x Ipsilateral | 1.1^**^ (.40) | .006 |
| Reaction time x Contralateral | -.39 (.58) | .50 |

Number of subjects: 22. Number of observations: 12645

# Appendix 3: Bayesian analyses

Bayesian analyses were implemented using the Brms package in R (Bürkner, 2016). Confidence was analysed with ordered logistic models, and accuracy was analyzed with Bernoulli models. In all cases, we used weakly informative priors (Normal(0,10)) for regression parameters, and performed 2000 iterations (burn-in period: 1000).

Table S3.1: Predicting confidence from accuracy, ispilateral and contralateral partial activation, reaction time and force production. Predictors were coded as follows – Accuracy: error = 0,, correct = 1; Ipsilateral : absent = 0, present = 1; Contralateral : absent = 0, present = 1. Ordered logit model, implemented with brms package in R.

| Predictor | β | lower 95% CI | upper 95% CI | Rhat |
| --- | --- | --- | --- | --- |
| Accuracy | 1.3 (.31) | 1.05 | 1.6 | 1.0 |
| Reaction time | -5.1 (.47) | -6.1 | -4.2 | 1.0 |
| Force production | .65 (.25) | .16 | 1.1 | 1.0 |
| Ipsilateral | .34 (.093) | .16 | .54 | 1.0 |
| Contralateral | .36 (.12) | .11 | .61 | 1.0 |

Number of subjects: 19. Number of observations: 11471

Table S3.2: Predicting confidence from accuracy, ispilateral and contralateral partial activation, reaction time force production and the interactions between partial activations and reaction time. Predictors were coded as follows – Accuracy: error = 0, correct = 1; Ipsilateral: absent = 0, present = 1; Contralateral: absent = 0, present = 1. Ordered logit model, implemented with brms package in R.

| Predictor | β | lower 95% CI | upper 95% CI | Rhat |
| --- | --- | --- | --- | --- |
| Accuracy | 1.2 (.16) | 1.04 | 1.7 | 1.0 |
| Reaction time | -5.4 (.52) | -6.5 | -4.4 | 1.0 |
| Force production | .59 (.24) | .12 | 1.1 | 1.0 |
| Ipsilateral | .19 (.11) | -0.27 | .41 | 1.0 |
| Contralateral | .28 (.13) | .022 | .55 | 1.0 |
| Reaction time x Ispilateral | 1.1 (.80) | .47 | 2.7 | 1.0 |
| Reaction time x Contralateral | 1.1 (.42) | .30 | 1.9 | 1.0 |

Table S3.3: Predicting confidence from accuracy, ispilateral and contralateral partial activation, reaction time , absolute pre-motor yime and force production. Predictors were coded as follows – Accuracy: error = 0, correct = 1; Ipsilateral: absent = 0, present = 1; Contralateral: absent = 0, present = 1. Ordered logit, implemented with brms package in R.

| Predictor | β | lower 95% CI | upper 95% CI | Rhat |
| --- | --- | --- | --- | --- |
| Accuracy | 1.3(.16) | 1.0 | 1.7 | 1.0 |
| Reaction time | -4.9 (.59) | -6.1 | -3.7 | 1.0 |
| Apmt | -.38 (.59) | -1.6 | .75 | 1.0 |
| Force production | .64 (.26) | .11 | 1.1 | 1.0 |
| Ipsilateral | .30 (.10) | .10 | .50 | 1.0 |
| Contralateral | .31 (.13) | .065 | .57 | 1.0 |

Number of subjects: 19. Number of observations: 11471

Table S3.4: Predicting accuracy from ispilateral and contralateral partial activation, reaction time, absolute pre-motor time and force production. Predictors were coded as follows – Ipsilateral: absent = 0, present = 1; Contralateral: absent = 0, present = 1. Ordered logit, implemented with brms package in R.

| Predictor | β | lower 95% CI | upper 95% CI | Rhat |
| --- | --- | --- | --- | --- |
| Intercept | 1.7 (.25) | 1.2 | 2.2 | 1.0 |
| Reaction time | -2.3 (.45) | -3.2 | -1.4 | 1.0 |
| Force production | .14 (.21) | -.27 | .59 | 1.0 |
| Ipsilateral | -.095 (.17) | -.42 | .24 | 1.0 |
| Contralateral | .17 (.17) | -.14 | .54 | 1.0 |

Table S3.5: Predicting accuracy from ispilateral and contralateral partial activation, reaction time, absolute pre-motor time, force production and the interactions between partial activations and reaction time. Predictors were coded as follows –Ipsilateral: absent = 0, present = 1; Contralateral: absent = 0, present = 1. Ordered logit, implemented with brms package in R.

| Predictor | β | lower 95% CI | upper 95% CI | Rhat |
| --- | --- | --- | --- | --- |
| Intercept | 1.7 (.27) | 1.2 | 2.3 | 1.0 |
| Reaction time | -2.4 (.48) | -3.3 | -1.4 | 1.0 |
| Force production | .12 (.21) | -.29 | .57 | 1.0 |
| Ipsilateral | -.38 (.19) | -.74 | .001 | 1.0 |
| Contralateral | .24 (.17) | -.074 | .59 | 1.0 |
| Reaction time x Ispilateral | 1.3 (.56) | .25 | 2.4 | 1.0 |
| Reaction time x Contralateral | -.25 (.72) | -1.6 | 1.2 | 1.0 |

Number of subjects: 19. Number of observations: 11471
